# Assessment of final year medical students knowledge of basic head and neck clinical anatomy

**DOI:** 10.1186/1753-6561-9-S1-A58

**Published:** 2015-01-14

**Authors:** J Al Amiri, MA Walsh

**Affiliations:** 1 Royal College of Surgeons in Ireland, 123 St. Stephen’s Green, Dublin 2, Ireland; 2 Otolaryngology Head and Neck Surgery, Royal College of Surgeons in Ireland, Beaumont Hospital, Dublin 9, Ireland

## Background

At the Royal College of Surgeons in Ireland (RCSI) anatomy teaching is carried out during the preclinical years by using various modalities to maximize students learning. The purpose of this study is to assess how much did final year student retain from the basic clinical anatomy of the head and neck and to determine if reinforcement of anatomy is required throughout the medical school curriculum.

## Methods

The study was carried out on 247 final year RCSI medical students.The students were asked to complete a multiple-choice quiz within 12 minutes. In addition, they were invited to fill in a short survey regarding their opinion on the anatomy curriculum.

## Results

The response rate to the quiz and survey were 64.78% and 55.56%, respectively. Out of a maximum score of 15, the mean score achieved was 7.58 and the mode was 9. Using the mode as our acceptable standard; 41.25% of the class passed the quiz. Students scored highly on neck surface anatomy questions, while scored low on questions related to cranial and peripheral nerves; cervical vertebra; and scalp injury. The majority of the responders to the survey felt that anatomy taught in the preclinical years was clinical relevant and that it should be reinforced throughout the clinical years.

## Conclusions

The knowledge of final year medical students of basic head and neck anatomy was acceptable, considering the time span between their preclinical and clinical years. However, the results highlight the need for reinforcement of relevant clinical anatomy throughout their clinical years of teaching.

